# Exosomes released by metabotropic glutamate receptor 1 (GRM1) expressing melanoma cells increase cell migration and invasiveness

**DOI:** 10.18632/oncotarget.23455

**Published:** 2017-12-19

**Authors:** Allison L. Isola, Kevinn Eddy, Krzysztof Zembrzuski, James S. Goydos, Suzie Chen

**Affiliations:** ^1^ Susan Lehman Cullman Laboratory for Cancer Research, Ernest Mario School of Pharmacy, Rutgers, The State University, Piscataway, NJ 08854, USA; ^2^ Joint Graduate Program in Toxicology, Rutgers, The State University, Piscataway, NJ 08854, USA; ^3^ Rutgers Cancer Institute of New Jersey, New Brunswick, NJ 08901, USA

**Keywords:** exosomes, GPCR, melanoma, GRM1, cancer

## Abstract

Exosomes are naturally occurring membrane-bound nanovesicles generated constitutively and released by various cell types, and often in higher quantities by tumor cells. Exosomes may facilitate communication between the primary tumor and its local microenvironment, supporting cell invasion and other early events in metastasis. A neuronal receptor, metabotropic glutamate receptor 1 (GRM1), when ectopically expressed in melanocytes, induces *in vitro* melanocytic transformation and spontaneous malignant melanoma development *in vivo* in a transgenic mouse model. Our earlier studies showed that genetic modulation in GRM1 expression by siRNA or disruption of GRM1-mediated glutamate signaling interfere with downstream effectors resulting in a decrease in both cell proliferation *in vitro* and tumor progression *in vivo*. In this study, we sought to determine whether exosome formation might play a role in GRM1 mediated melanoma development and progression. To test this, we utilized *in vitro* cultured cells in which GRM1 expression and function could be modulated by pharmacological and genetic means and determined effects on exosome production. We also tested the effects of exosomes from GRM1 expressing melanoma cells on growth, migration and invasion of GRM1 negative cells. Our results show that although GRM1 expression has no influence on exosome quantity, exosomes produced by GRM1-positive cells modulate the ability of the recipient cell to migrate, invade and exhibit anchorage-independent cell growth.

## INTRODUCTION

Melanoma patients only account for about 5% of all skin cancer cases, but it is the subset that accounts for the majority of deaths [1]. Metabotropic glutamate receptor 1 (GRM1) is a seven transmembrane-domain G-protein coupled receptor (GPCR) that, upon activation by ligand binding, initiates signaling cascades resulting in the downstream activation of the MAPK signaling cascade, involved in cell proliferation and inhibition of apoptosis [2, 3], and the PI3K/AKT pathway [4–7], involved in tumor cell survival, epithelial-mesenchymal transition and angiogenesis [8, 9].

Our laboratory showed that a gain-of-function of murine GRM1, when ectopically expressed in melanocytes, induced *in vitro* melanocytic transformation and spontaneous malignant melanoma development *in vivo* in transgenic mouse models with 100% penetrance [10–14]. Exogenous GRM1 was introduced into human melanoma cell lines with either modest GRM1 expression or absence of detectable GRM1 expression, and showed that enhanced GRM1 expression levels led to upregulated angiogenesis and increased tumorigenesis *in vitro* and *in vivo* [15].

Subsequent studies revealed GRM1 RNA and protein overexpression in 80% of human melanoma cell lines and 65% of human melanoma biopsy samples [14]. GRM1 RNA or protein were not detectable in normal melanocytes [16]. Additionally, levels of elevated glutamate, the natural ligand of GRM1, were found only in GRM1-expressing melanoma cells [17], suggesting the establishment of an autocrine loop. Consistent with this, exposure to GRM1 antagonists led to reduced melanoma cell growth *in vitro* and tumorigenicity *in vivo* [12, 17]. Finally, riluzole, an FDA approved drug for Amyotrophic Lateral Sclerosis, which inhibits the release of glutamate, also led to a decrease in melanoma cell growth *in vitro* and tumor progression *in vivo*. Similar observations have been made in breast [18] and prostate cancer cells [14, 17] that were shown to express GRM1.

Exosomes are small membrane-bound nanovesicles that play many different roles in normal physiology [19]. In cancer, exosomes have been shown to contribute to the essential cancer hallmarks, namely: sustaining proliferative signaling, evading growth suppressors, resisting cell death, enabling replicative immortality, inducing angiogenesis, promoting genome instability and mutations, increasing tumor-promoting inflammation and especially activating invasion and metastasis [20]. These microvesicles are more frequently released by tumor cells and may facilitate communication between the primary tumor and its local microenvironment [21–24]. Exosomes may have the ability to promote metastasis via the horizontal transfer of proteins, miRNAs and other molecules to recipient cells [25–28].

Preliminary studies showed that elevated levels of exosomes correlated with an increase in GRM1 expression (Goydos and colleagues, data unpublished), prompting the hypothesis that the glutamatergic signaling cascades in GRM1 expressing melanoma cells may mediate their tumorigenic effect in part through exosome production and secretion.

## RESULTS

### Comparisons between ultracentrifugation/sucrose gradient and a commercial kit in the isolation of exosomes

**Figure 1:**
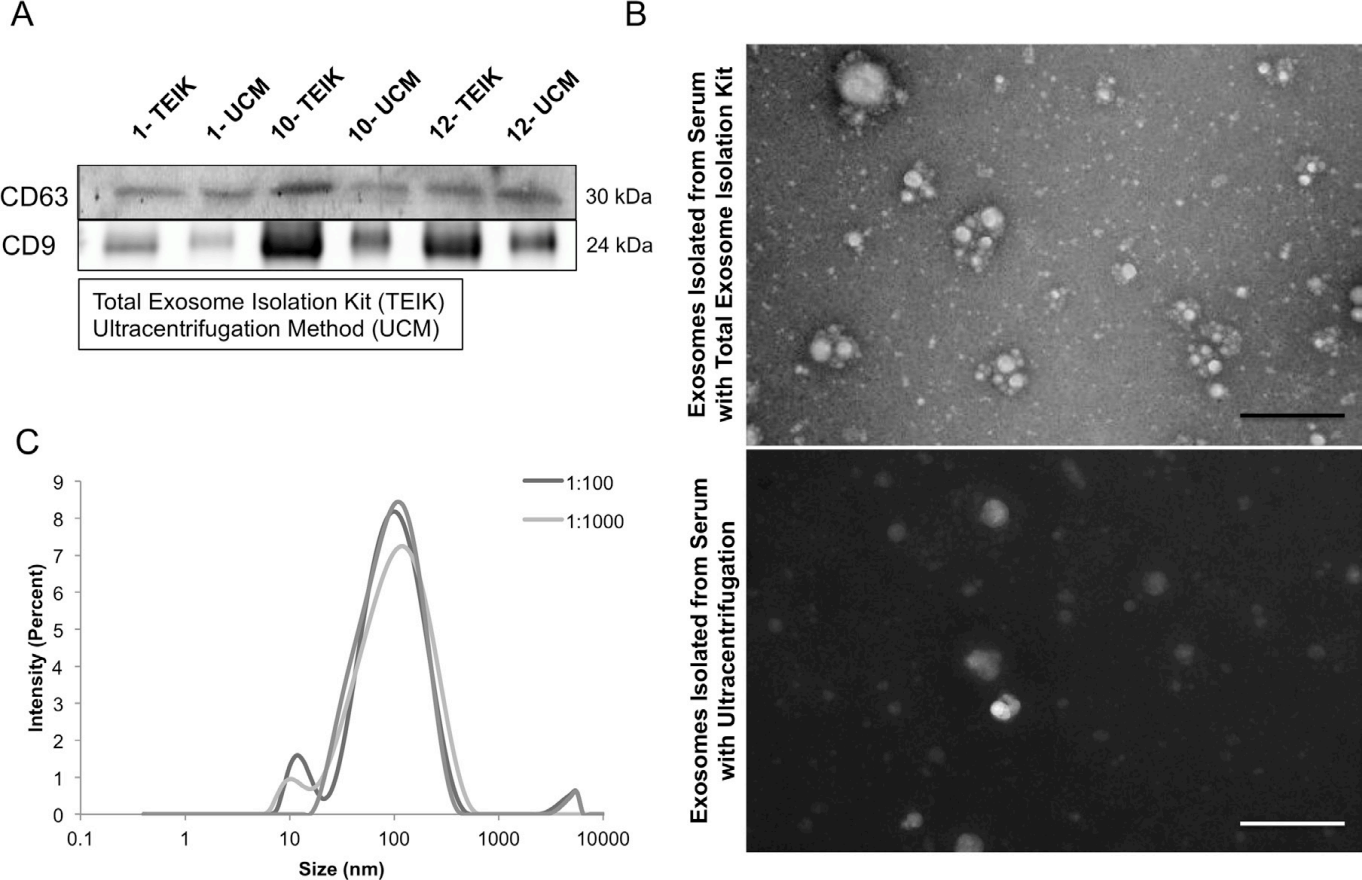
Exosome isolation method Representative CD63 immunoblot for serum exosome lysates (SEL) from identical volumes of serum from untreated SKH-1 mice, where the Total Exosome Isolation Kit (Invitrogen) (TEIK) was compared to the Ultracentrifugation Method (UM). Number indicates mouse identification number **(A)**. Representative electron micrographs of samples isolated by either the total exosome isolation kit or ultracentrifugation showed intact exosomes of the correct size in both samples. Although, a higher level of background protein staining was observed in the samples isolated with the ultracentrifugation method, as seen here by the dark background, the scale bar is 500nm **(B)**. Serum exosome samples were ran on the Zetasizer to determine the particle sizes present, each curve representing various dilutions **(C)**.

Many different methods are used to isolate and quantify exosomes, including ultracentrifugation through sucrose gradients and commercial kits. To determine which approach would best serve this project, a side-by-side comparison was performed between the Total Exosome Isolation Kit (TEIK) and the commonly used method of ultracentrifugation (UCM). Mouse blood samples were taken from 3 different animals (SKH-1 mouse #1, 10 and 12) and exosomes were isolated from these blood plasma samples. CD63, a protein enriched in exosomes, and commonly used as an exosome marker [29–31], as well as an alternative exosome enriched protein, CD9, were analyzed in the samples by immunoblots. The results show that both markers are present in exosome isolates from both methods, and in some cases are enriched in the total exosome isolation kit samples (Figure 1A). To assess the quality of the exosome preparation, electron microscopy was performed. Intact exosomes of the characteristic size were found in both ultracentrifugation and kit methods. One difference observed in the 2 methods was a black background present in the ultracentrifugation samples, indicating a higher soluble protein contamination when compared to the exosomes isolated using the kit (Figure 1B). These data indicate that the commercial kit, in some cases, enriches for a higher quantity of CD63-protein rich exosome fractions with minimal protein contamination when compared to the commonly used ultracentrifugation isolation method.

The Zetasizer Nano (Malvern) was used to measure the size of the particles present in the exosome suspension. It utilizes Brownian motion principles to measure the diffusion of particles and their motion, converting it to a size distribution using the Stokes-Einstein relationship. Back Scatter technology is used to give the high sensitivity measurement of size and concentration. Figure 1C shows the size distribution of the particles in the exosome isolation suspension with a peak around 100nm; consistent with the characteristic size of exosomes (30-120nm).

### Ectopic GRM1 expression in C81-61 cells shows little effect on exosome release

To determine if GRM1 affects the levels of released exosomes, two complementary approaches were used. The first involved the introduction of exogenous human GRM1 cDNA into an early stage melanoma cell line, C81-61, which does not express endogenous GRM1. *In vitro* and *in vivo* characterization of several GRM1-expressing C81-61 clones showed these clones are now transformed and tumorigenic [15]. Here we selected C81-61-GRM1-6 for further studies. Exosome levels were compared between the parental C81-61 and C81-61 GRM1 clones.

**Figure 2:**
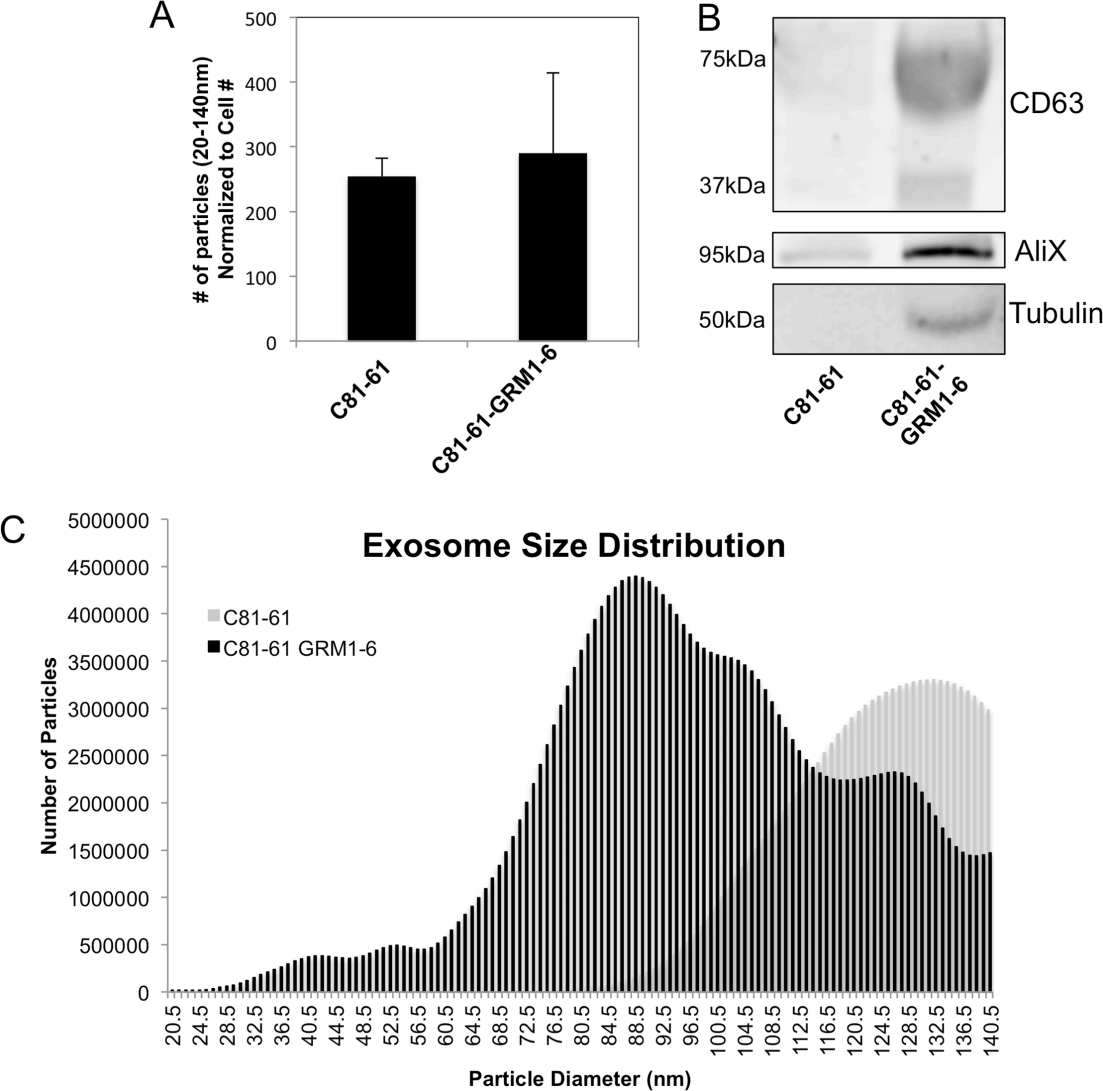
GRM1 expression results in changes in exosome size distribution Nanosight quantification shows no change in exosome number isolated from C81-61-GRM1-6 when compared to C81-61 and normalized to cell number **(A)**, however, when normalized to cell number, the difference in exosome number is negligible. Immunoblots showed an increase in exosome protein markers in C81-61-GRM1-6 when compared to the parental C81-61, however, when normalized to tubulin, the increase is dampened to an insignificant amount, sometimes the molecular weight of glycosylated form of CD63 may range from 30-60 kDa **(B)**. Nanosight analysis indicates a shift in size of exosomes released by cells expressing GRM1. Exosomes isolated from C81-61-GRM1-6 conditioned media showed a smaller average size when compared to the parental C81-61 exosomes **(C)**.

C81-61 and C81-61-GRM1-6 cells were plated, incubated overnight, the media were then replaced with serum-free OptiMEM media and incubated for an additional 48 hours. OptiMEM media was used to avoid possible contamination from exosomes present in the serum used in standard culture media. The exosomes were isolated from conditioned cell culture media and quantified using the Nanosight. The results show no significant change in number of exosomes released by C81-61-GRM1-6 cells when compared to the parental C81-61 on a per cell basis (Figure 2A). Two exosomal markers (CD63, AliX) and an internal standard (tubulin) were also used in western immunoblots to assess exosomal levels. Band intensity was greater in the exosome protein samples in C81-61-GRM1-6 samples compared to the parental C81-61 cells, but the increase was not significant when normalized to tubulin concentration (Figure 2B).

### Alterations in size distribution of exosomes in cells with GRM1 expression

Particle size analysis was performed using the Nanoparticle Tracking Analysis (NTA) software on exosomes isolated from C81-61 and C81-61-GRM1-6 cells. A smooth unimodal distribution of exosome size secreted by C81-61 cells was detected. In contrast, exosomes isolated from C81-61-GRM1-6 cells contained a large number of smaller, more heterogeneous vesicles in addition to the exosomes of similar size distribution to C81-61 (Figure 2C).

### Genetic modulation of GRM1 expression in cells did not affect release of exosomes

**Figure 3:**
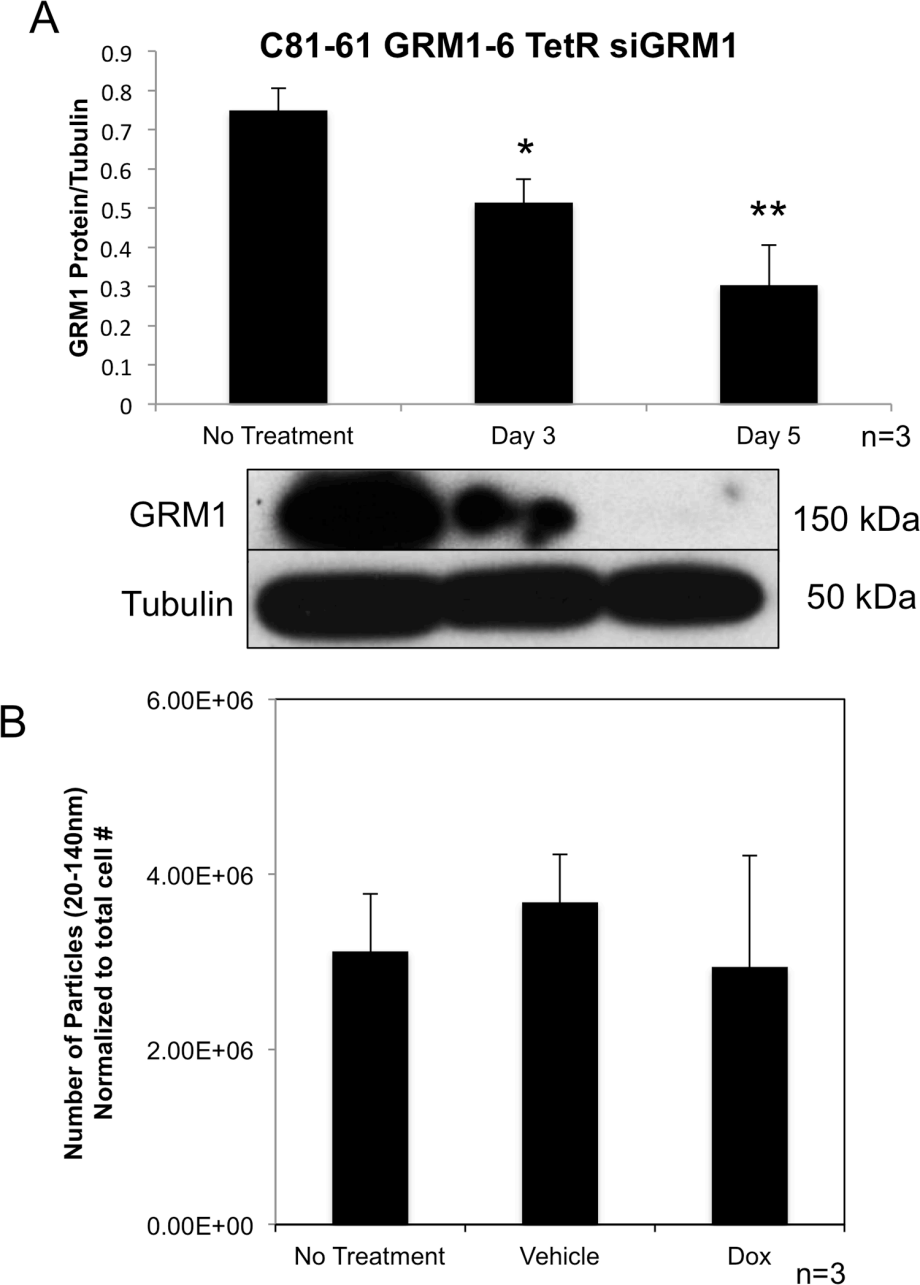
Exosome levels are unchanged with varying levels of GRM1 protein Representative western blot quantification showing a reduction in GRM1 protein with treatment of doxycycline (10ng/ml)(p<0.01) **(A)**. Nanoparticle Tracking Analysis of exosomes isolated from the conditioned media of C81-61 GRM1-6 TetR siGRM1 cells after 48 hours **(B)**.

In order to determine if the level of GRM1 protein present within the cells affects the amount of exosomes released by the cells, we took advantage of the inducible Tet-On silencing RNA system to modulate GRM1 expression levels in C81-61-GRM1-6 cells. C81-61-GRM1-6 cells were transfected with both TetR and siGRM1 plasmids to create several C81-61-GRM1-6-TetR-siGRM1 clones, clone 16 was selected for further characterization. In the presence of the inducer, doxycycline, the amount of GRM1 was reduced substantially as shown by the immunoblot (Figure 3A).

We then isolated exosomes from cultured cells with or without the inducer, doxycycline and analyzed them with the Nanosight. No alteration was seen in exosome number when normalized to cell number and compared to vehicle or no treatment controls (Figure 3B).

### Pharmacological modulation of GRM1 function did not affect exosome release by melanoma cells

In addition to using genetic means to modulate GRM1 expression, pharmacological glutamate signaling blockades were used in these *in vitro* approaches. Two types of blockades were used: first, an inhibitor of glutamate release, riluzole, an FDA approved drug for treatment of Amyotrophic Lateral Sclerosis (ALS). The ability of riluzole to block the release of glutamate and subsequently reduce levels of available ligand allows it to act functionally as an inhibitor of GRM1-mediated signaling and interferes with intracellular events that follow stimulation of the receptor. The second pharmacological reagent was the specific non-competitive inhibitor of GRM1, Bay36-7620, which binds the intracellular loops and alters the conformation of the receptor rendering it non-functional. Both blockades were shown previously by our group to reduce cell growth *in vitro* and tumor progression *in vivo* [17].

**Figure 4:**
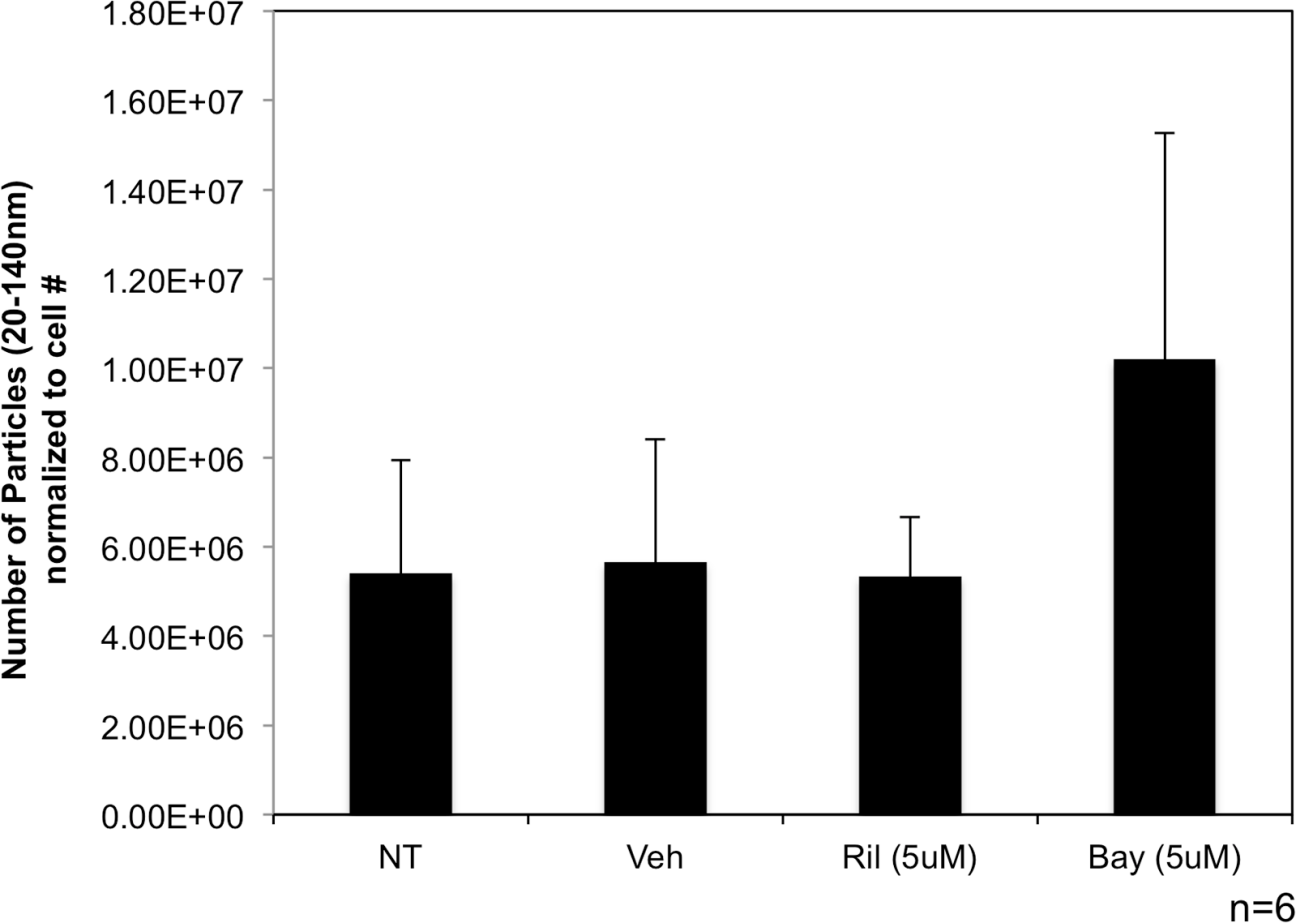
Exosome levels are unchanged when treated with GRM1 inhibitors Western blot showing expression of GRM1 protein in C81-61 cells transfected with GRM1 **(A)**. Nanosight analysis of exosomes isolated from C81-61-GRM1-6 cells treated with either riluzole or Bay36-7620 **(B)**.

C81-61-GRM1-6 cells were treated with riluzole (5μM) or Bay36-7620 (5μM) for 48 hours. Nanosight quantification was used to determine the number of nanovesicles secreted into the media. We found no significant change in the number of exosomes released from either riluzole or Bay36-7620 treated cells (Figure 4) when normalized to cell number and compared to untreated and vehicle controls.

**Figure 5:**
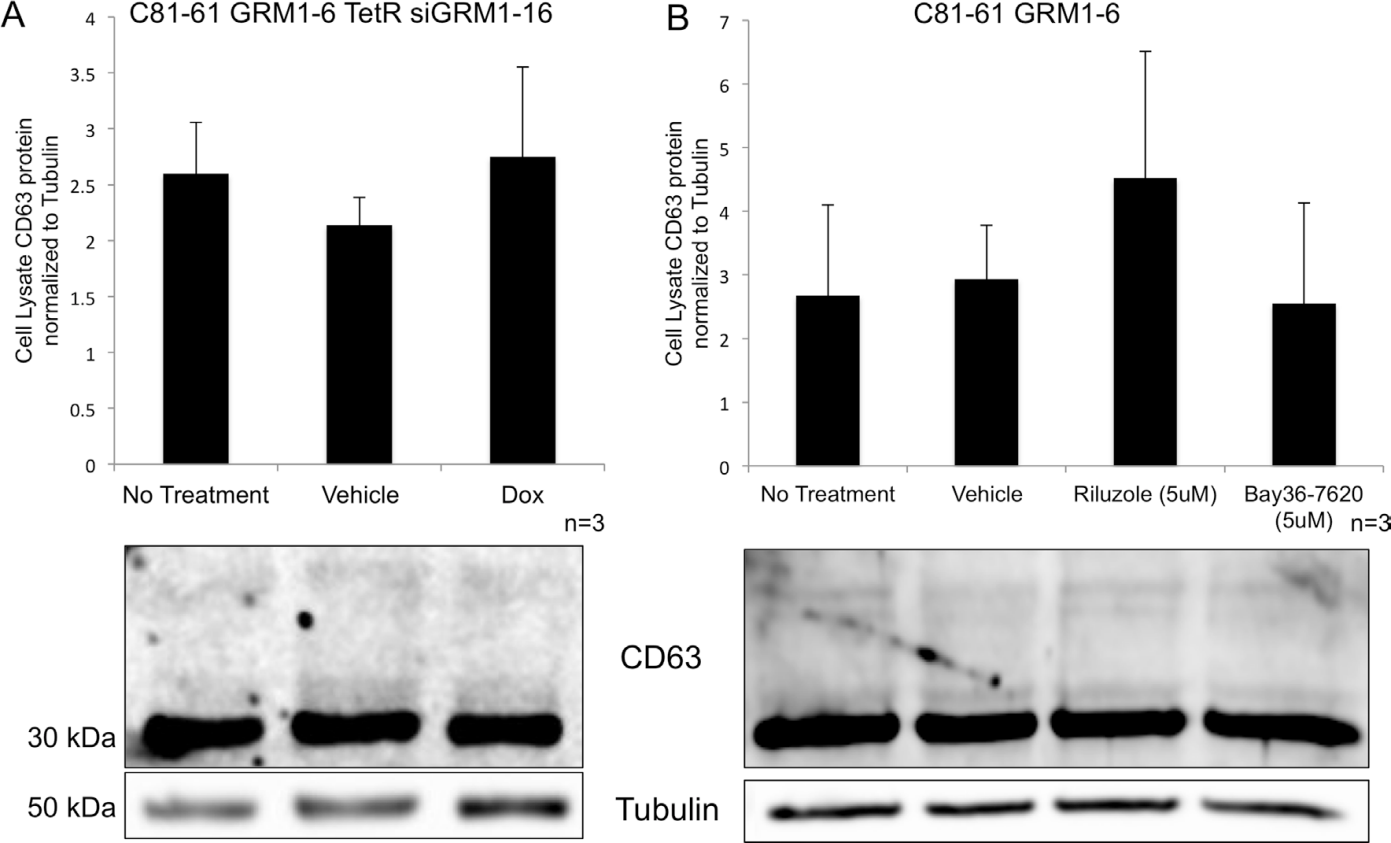
Levels of intracellular CD63 protein in melanoma cells are unaffected by treatment Immunoblots for CD63 protein from the cell lysates of melanoma cells with various treatments and normalized to tubulin protein levels. C81-61-GRM1-6 TetR siGRM1 – 16 cells were treated with vehicle control (DMSO) or doxycycline and no differences were seen in the amount of CD63 protein present in the cell **(A)**. C81-61-GRM1-6 cells were treated with vehicle control (DMSO), 5μM riluzole or 5μM Bay36-7620, and no differences were observed in the CD63 protein levels **(B)**.

We also evaluated CD63 expression in cells where the GRM1 expression (by silencing RNA) or function (by pharmacological inhibitors) has been modulated. No change in the level of the exosomal protein marker, CD63, was seen within the cells when normalized to tubulin as a loading control (Figure 5A and 5B).

### Exosomes from GRM1^+^ cells do not promote cell proliferation in GRM1^-^ cells

C81-61 cells were treated with either C81-61 or C81-61-GRM1-6 conditioned media. Cellular proliferation was measured using a colorometric cell proliferation/cell viability assay. Comparisons were made between C81-61 incubated in the untreated control media, conditioned media from C81-61 cells and conditioned media from C81-61-GRM1-6, very similar growth rate was observed in C81-61 cells incubated in all three media. These results indicate that exosomes released from C81-61-GRM1-6 in the conditioned media did not promote cell proliferation in C81-61 cells (Figure 8A).

**Figure 6:**
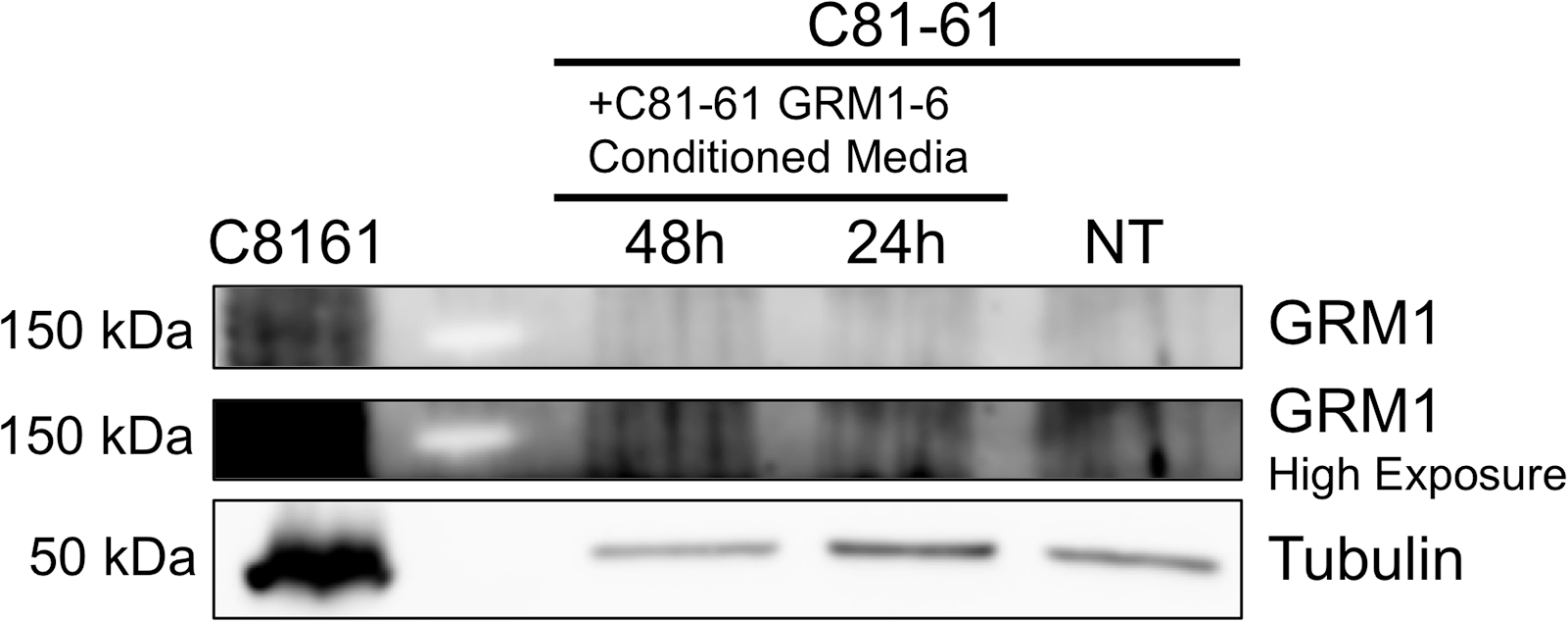
Undetectable transfer of GRM1 protein from exosomes to recipient cell Western blot with GRM1 antibody of washed cell lysates of C81-61 cells incubated with conditioned media from C81-61 GRM1-6 cells.

**Figure 7:**
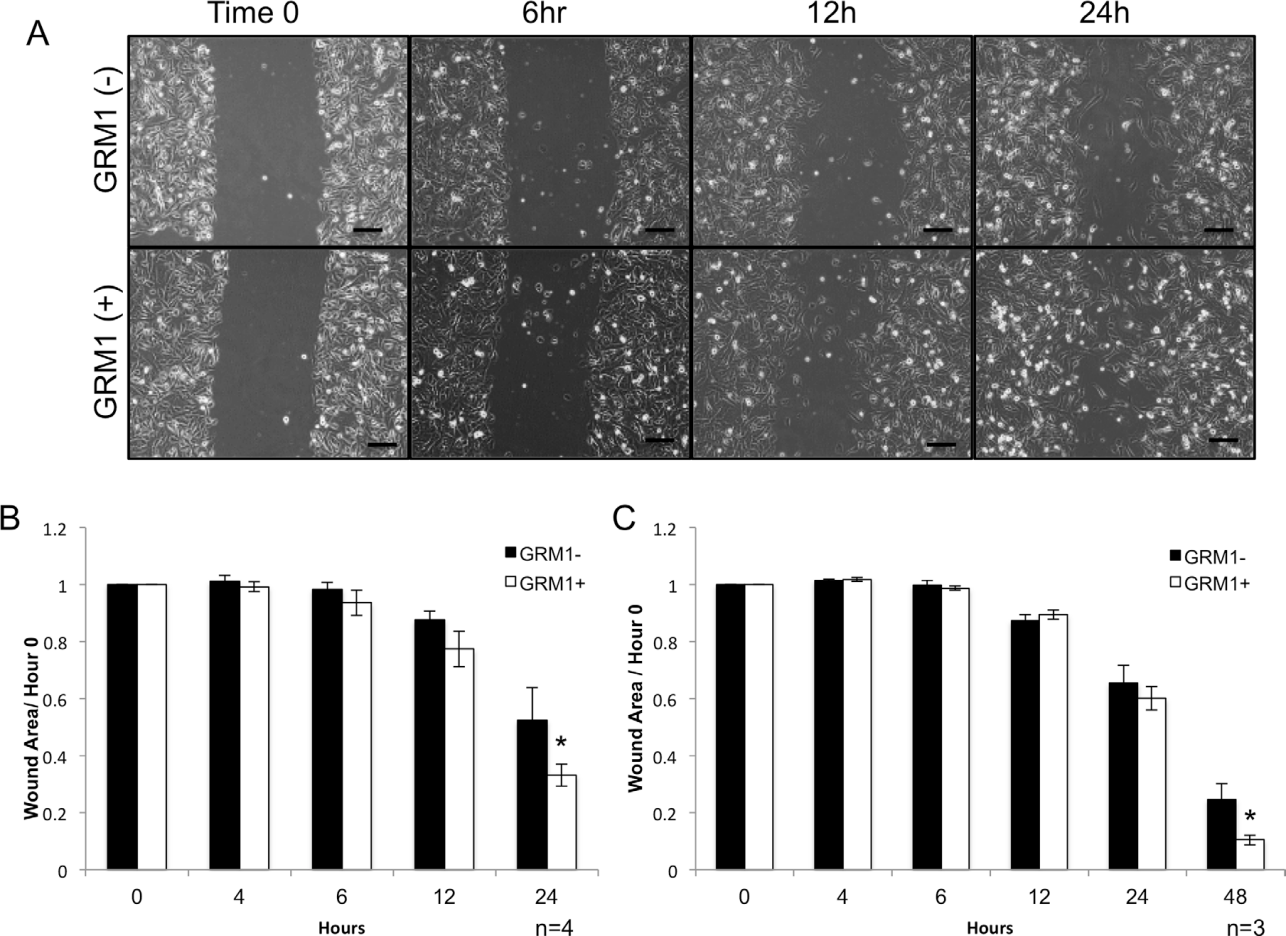
GRM1^-^ cells exhibit increased mobility when exposed to GRM1^+^ cell derived exosomes C81-61 melanoma cells (GRM1^**-**^) were incubated with conditioned media from either C81-61 (GRM1^**-**^) cells or C81-61 GRM1-6 (GRM1^**+**^) cells, a scratch was made in the confluent cell layer, and photographs were taken at various time points, the scale bar is 100μm **(A)**. Wound area was calculated using ImageJ, and normalized to the size of the original wound (Time 0). A significant reduction in wound size was observed in the cells incubated with exosomes derived from C81-61-GRM1-6 (p=0.02, n-4) **(B)**. Wound healing assay was also performed using C81-61 cells incubated with purified exosomes from either C81-61 (GRM1^**-**^) cells or C81-61-GRM1-6 (GRM1^**+**^) cells. After 48 hours post-wound, a significant reduction in wound area was seen in the cells incubated with purified exosomes derived from C81-61-GRM1-6 (GRM1^**+**^) cells (p=0.014, n=3) **(C)**.

**Figure 8:**
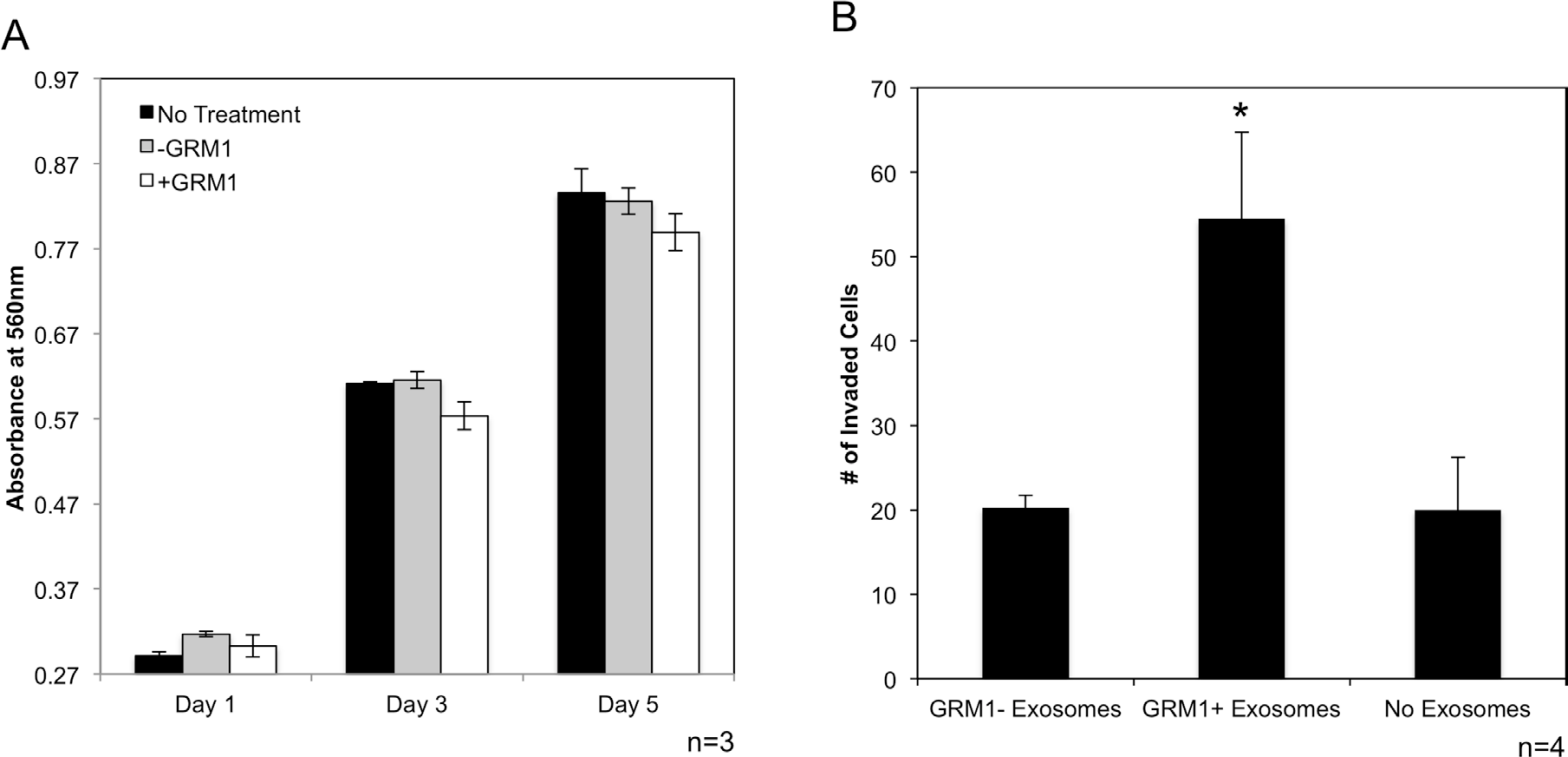
GRM1+ exosomes induce invasion in GRM1- cells C81-61 cells were incubated with conditioned media from C81-61 or C81-61 GRM1-6 cells and cell proliferation was measured using the MTT cell proliferation assay. Cell proliferation was unaffected by conditioned media from the 2 cell lines on Day 1, 3 and 5 of incubation **(A)**. Migration of C81-61 cells incubated with exosomes released from C81-61 cells (GRM1- exosomes), C81-61 GRM1-6 cells (GRM1+ exosomes) or no exosomes. Results of the representative experiment shows the number of cells in 10 random fields. The number of cells invaded when incubated with exosomes from C81-61 GRM1-6 is significantly higher than those incubated with exosomes from C81-61 cells (p=0.016) **(B)**.

### Exosomes from GRM1^+^ cells induce migration in GRM1^-^ cells

Earlier reports from other investigators demonstrated that exposure to exosomes from metastatic cells altered the behavior of non-metastatic cells, including increasing their metastatic capability [32, 33]. We performed wound healing assays to assess possible differential migration abilities of C81-61 incubated with media conditioned by either GRM1^+^ (C81-61-GRM1-6) or GRM^–^ (C81-61) cells. After a 24-hour incubation with GRM1^+^ conditioned media, C81-61 cells exhibited a significant increase in migration into the culture wound (p<0.05) (Figure 7A and 7B).

We then assessed if this increased migration property is a result of exosomes released into the growth media, we isolated and purified exosomes from the conditioned media and repeated the experiment. C81-61 (GRM1^-^) cells were incubated with exosomes from either GRM1^+^ (C81-61-GRM1-6) or GRM^–^ (C81-61) cells for 24 hours and the cultures were scraped. Images were captured at different time points and the wound healing analysis showed a similar increase in migration induced by GRM1^+^ exosomes as shown in conditioned media from GRM1^+^ (C81-61-GRM1-6) cells, except for purified exosomes it took 48 hours instead of 24 hours for conditioned media (p<0.005) (Figure 7C).

### Exosomes from GRM1^+^ cells induce invasion in GRM1^-^ cells

In order for any tumor cell to metastasize, the cell must have the ability to invade the surrounding tissue, embed and proliferate in distant tissues in the body. We therefore determined if C81-61 cells acquire an invasive property when incubated with exosomes from GRM1^+^ cells, using an *in vitro* invasion assay. Exosomes from C81-61-GRM1-6 and C81-61 cells were isolated from cell culture media after 48 hours. C81-61 cells were incubated with exosomes from either C81-61 or C81-61-GRM1-6, or no exosomes for 2 hours, and then seeded on the matrigel invasion chamber plate. After a 72-hour incubation, cells that had migrated through the matrigel were stained and counted in 10 random fields. A significant increase in the number of cells migrated after incubation with GRM1^+^ cell exosomes was seen when compared to those cells incubated with either no exosomes or exosomes from GRM1^-^ cells (Figure 8B).

### Confirmation of generated cell lines

**Figure 9:**
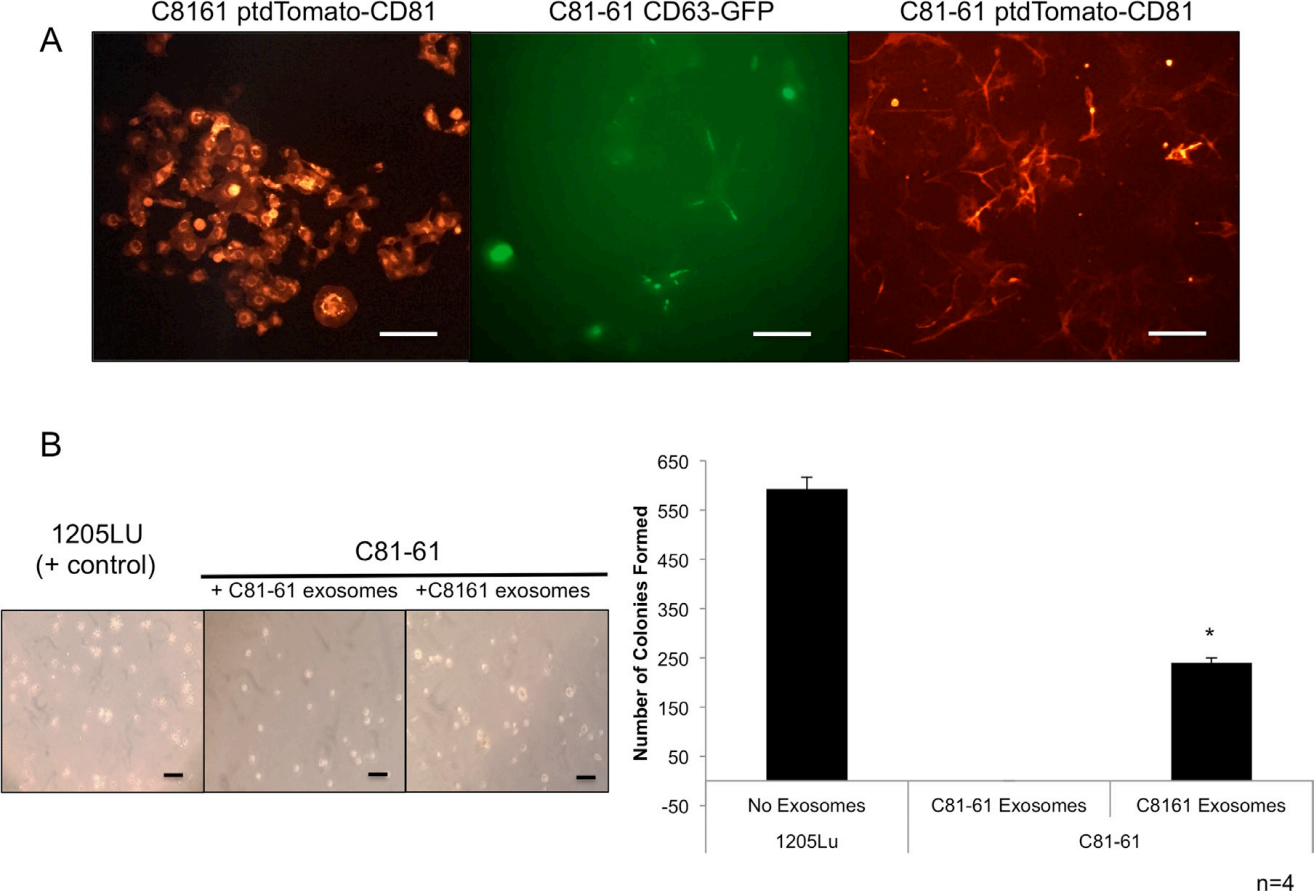
GRM1+ exosomes induce anchorage independent growth in GRM1- cells C81-61 cells were stably transfected with either CD63-GFP or ptdTomato-CD81. The Keyence BZ-X710 florescent microscope was used to confirm the presence of GFP or ptdTomato florescent tags in stably transfected C8161 ptdTomato-CD81, C81-61 CD63-GFP, and C81-61 ptdTomato-CD81, the scale bar is 100μm **(A)**. Cells were photographed after 21 days of growth in soft agar and exosomes. 1205Lu serves as a positive control. C81-61 CD63-GFP cells were plated in media with 0.33% agarose and incubated with either C8161 ptdTomato-CD81 exosomes that show significant number of colony formation, or with C81-61 ptdTomato-CD81 exosomes that only show two colonies (n=4, t-test, p=0. OT_031814_proof5), the scale bar is 100μm **(B)**.

C81-61 CD63-GFP, C8161 ptdTomato-CD81 and C81-61 ptdTomato-CD81 stable clones were viewed under fluorescent microscope for the presence of the GFP or ptdTomato fluorescent tags within the cells (Figure 9A).

### Exosomes from GRM1^+^ cells induce anchorage-independent colony formation in GRM1^-^ cells

The soft agar colony formation assay is used to determine the ability of cells to form colonies without the dependence on an extracellular matrix contact. C81-61 CD63-GFP cells were grown in a layer of soft agar containing medium with either exosomes isolated from C81-61 ptdTomato-CD81 cells or C8161 ptdTomato-CD81. The tumorigenic human melanoma cell line, 1205Lu, was used as a positive control. Cells were fed with a fresh agarose containing medium and exosomes once a week, and after 21 days, the number of colonies formed were quantified. C81-61 CD63-GFP cells incubated with exosomes isolated from C8161 ptdTomato-CD81 cells formed a significantly higher number of colonies when compared to C81-61 CD63-GFP cells with exosomes isolated from C81-61 ptdTomato-CD81 cells (Figure 9B).

### Exosomes from GRM1^+^ cells do not transfer GRM1 protein to GRM1^-^ cells

To explore the possible mechanism of the induction of migration and invasion abilities in C81-61 cells by C81-61-GRM1-6 exosomes, C81-61 cells were incubated with the conditioned media from C81-61-GRM1-6 cells. The C81-61 cells were then washed multiple times and protein was extracted. An immunoblot for GRM1 was performed, and α-tubulin was used as a loading control. Immunoblot results indicate the absence of GRM1 in cells treated with C81-61-GRM1-6 conditioned media while a band was present in the positive control (Figure 6). These data indicate GRM1 protein is not transferred from the exosomes to the recipient cells to induce metastatic abilities.

## DISCUSSION

In this study, the role of metabotropic glutamate receptor 1 (GRM1) in the production and release of exosomes in melanoma cells was explored. The transfection of GRM1 cDNA into the C81-61 non-tumorigenic melanoma cell line results in its transformation into an aggressive, tumorigenic cell line [15]. Although different groups have shown an increase in exosome release by aggressive, tumorigenic cell lines, when compared to their non-tumorigenic or normal counterparts [21–24], our results using several established exosome quantification methods did not show a significant difference between the non-tumorigenic C81-61 and the tumorigenic C81-61-GRM1 clones. Similarly, when the expression of GRM1 was reduced (via inducible siGRM1) or the function of the receptor was blocked (via pharmacological inhibition by a non-competitive inhibitor, Bay36-7620, and a functional inhibitor, riluzole) no significant change in the number of exosomes released by treated cells was observed.

However, we did find that C81-61-GRM1 derived exosomes are functionally different as shown by significantly increased *in vitro* properties indicative of malignant behavior: migration and invasion and the ability to form colonies. Previous studies by others had similar observations that exosomes from aggressive tumorigenic lines can transfer such properties to less aggressive cells. Exosomes may induce these altered behaviors by transferring macromolecules from the originating cell to the recipient cells [25–28]. Melanoma cell derived exosomes have been shown to contain various determinants found in the cells from which they are derived. Several unique proteins only found in exosomes isolated from highly metastatic melanoma cell lines; these proteins play various roles in cell motility, suggesting that these exosomes have the capability of transferring pro-migratory proteins to the less aggressive cell lines [34]. Additionally, exosomes containing a known marker of poor outcome for ovarian cancer (RNA-binding protein, LIN28) have been shown to be present in recipient cells, resulting in the increase in production of proteins involved in epithelial-to-mesenchymal transition (EMT), cell migration and invasion [35].

In addition to the ability of exosomes to promote the pro-metastatic behavior of migration, normal melanocytes become invasive when exposed to exosomes from melanoma cells [36]. Highly metastatic of B16 melanoma cell (B16-10) derived exosomes have been shown to contain a pro-metastatic protein, Met72. When these exosomes are taken up by the poorly metastatic clone of B16, B16-F1, the recipient cells express of Met72 and adopt the metastatic behavior of the aggressive B16-10 cells [37].

We showed earlier that introduction of GRM1 into C81-61 resulted in cell transformation *in vitro*, tumor formation *in vivo* [15] and now we present evidences that the functionalities but not the overall levels of exosomes from GRM1-expressing cells were also altered and were able to promote an increase in migration and invasion when co-incubated with cells lacking such ability.

Preliminary proteomic analysis by mass spectrometry of exosomes isolated from the two isogenic cell lines, C81-61 and C81-61 GRM1-6 indicates an approximate 20% difference in the number of identified proteins present in exosomes between these two lines (data not shown). Exosomes from GRM1^+^ cells contain over 500 additional proteins when compared to the exosomes derived from GRM1^-^ cells, however exosomes isolated from GRM^-^ cells contain 200 proteins that were not present in the exosomes from GRM1^+^ cells. Based on this data, we speculate that the transfer of aggressive phenotype by exosomes from GRM1^+^ cells could be a result of the difference in either protein or RNA cargo delivered to the recipient cells. This observation of a difference in exosomal proteins could account for an increase in number of proteins involved in cell migration, invasion and potentially metastasis, delivered to recipient cells, while proteins involved in apoptosis or growth suppression could be present in those exosomes isolated from GRM1^-^ cells. We are currently in the process of analyzing and prioritizing these differentially expressed proteins and working to validate the potential players in altered cell behavior. In parallel, miRNA analysis is also ongoing and again potential candidates will be confirmed and validated by genetic approaches.

The aggressiveness and malignancy exhibited by cancers aberrantly expressing GPCRs, such as GRM1, may be due to the release of exosomes that are functionally more aggressive. The precise mechanisms of alterations of the exosomal pathway due to GRM1 activation and subsequent signaling remain unknown. This association between GRM1 and the aggressiveness of the exosomes released by cells expressing GRM1 may provide hints to elucidate the aggressive nature of GRM1-expressing melanomas, and the role of exosomes in their metastatic potential.

## MATERIALS AND METHODS

### Cell lines

C81-61 is a cell line derived from an early stage melanoma that is negative for GRM1 expression. Exogenous human GRM1 cDNA cloned in a mammalian expression vector was introduced into this cell line. Several stable clones were isolated and clone C81-61-GRM1-6 was selected for further characterization.

Silencing RNA to GRM1 in an inducible tetracycline regulated vectors were introduced into the C81-61-GRM1-6 cell line to allow modulation of GRM1 expression, we then assessed the effects of decreased production of GRM1 on exosome production. Specifically, C81-61-GRM1-6 cells were infected overnight with TetR lentiviral particles and 7.5 mg/mL polybrene (Millipore cat#TR-1003-G). Stable C81-61-GRM1-TetR cells were selected with blasticidin at 5 μg/ml. C81-61-GRM1-TetR cells were transfected using DOTAP reagent (Roche cat#11 811 177 001) with 4 μg of siGRM1 plasmid DNA cloned into the pRNATin-H1.2/Hygro vector as described [12]. C81-61-GRM1-TetR-siGRM1 clones were generated by double selection with 5 μg/ml blasticidin and 5 μg/ml hygromycin.

### Cell culture method

Cell lines were grown in RPMI 1640 medium with 10% Fetal Bovine Serum and 1% Penicillin/Streptomycin until confluent. 4x10^5^ C81-61-GRM1-6 cells were plated in serum free Opti MEM (Life Technologies cat#31985062) in 60mm cell culture dishes. Experimental groups included no treatment (NT), vehicle (DMSO), 5 μM riluzole and 5μM Bay36-7620 for 48 hours.

For induction studies using C81-61-GRM1-6-TetR-siGRM1-16, 4x10^5^ cells were plated in 60mm cell culture dishes in serum free Opti MEM, and treated with 10 μg/mL of doxycycline (concentration sufficient to induce siGRM1 production) for 48 hours after which exosomes are isolated.

To collect exosomal proteins for immunoblotting, C81-61 or C81-61-GRM1-6 cells were plated at 2.5×10^6^/plate in 4-150mm plates. After 24 hours, plates were rinsed with sterile 1X PBS, and media were changed to RPMI with 2% exosome-depleted fetal bovine serum (Gibco Ref#A25904DG), and exosomes collected after 48 hours.

### Cell lysate protein extraction

Culture media was aspirated and cells were washed twice with cold 1X PBS, and 600 μL of 10:1 Laemmli Sample Buffer: β-mercaptoethanol mixture was added to each 150 mm plate. The cells were then scraped and collected in a centrifuge tube. The samples were heated for 10 minutes at 99°C and then centrifuged at 15,000 x g for 10 minutes at room temperature. The supernatant containing the cell lysate was then transferred into a new tube to be analyzed by immunoblot.

### Immunoblot

Lysates were electrophoresed on 10% SDS gels after denaturation at 95ºC for five minutes. A reference protein ladder (Precision Plus Protein Standards-Bio-Rad Cat# 161-0374) was used to determine the size of the band. Gels were electrophoresed for two hours at 120 volts; proteins on the gel were transferred onto a nitrocellulose membrane (GVS North America Cat#1215471) for three hours at 160 mA. The membrane was then blocked using 0.25% milk (nonfat dry milk and 50 mM Tris-Cl, pH 7.6, 150 mM NaCl, 0.1% Tween-20) with a SnapID 2.0 (Millipore), to reduce nonspecific binding. The membrane was incubated overnight with its respective primary antibody: CD63 primary antibody (1:1,000, 5% BSA, 0.1% NaAz, Biorbyt Cat# orb13317), monoclonal AliX primary antibody (1:1,000, Cell Signaling Technology Cat# 2171S) or monoclonal anti-ɑ-tubulin antibody (Sigma Cat#T6074-200ul). After incubation, the membrane was washed five times on the SnapID 2.0 with wash buffer (1X TBS, 0.1% Tween-20). The blot was then incubated on a rocker for one hour at room temperature with the respective secondary antibody, either anti-rabbit (1:5,000, Dky x Rb IgG, Millipore Cat# AP182P) or anti-mouse IgG (1:5,000, Sigma Cat# A4416-1ML) in 0.25% w/v milk (1X TBS, 0.1% Tween-20). The blot was washed in the same manner as above, incubated with Forte Western HRP Substrate (Millipore Cat# WBLUF0100) for 3 minutes, and visualized using the GeneSys imager (Syngene). The band intensities were quantified using ImageJ computer software.

### Exosome isolation method

Conditioned cell culture media were concentrated up to 80-fold using a centrifugal filter (Millipore Centricon Plus-70 Ref#UFC710008). The Invitrogen Exosome Isolation kit (for cell culture media, Cat# 4478359) was used following manufacturer’s instructions. Briefly, concentrated cell culture media were centrifuged at 2,000 x g for 30 minutes at room temperature to remove cellular debris. Invitrogen Exosome Isolation Buffer for cell culture media was then added to the supernatant at a volume of 0.5 times the total cell culture media volume and incubated at 4°C overnight. The samples were then centrifuged for 1 hour at 10,000 x g creating a concentrated exosome pellet. The supernatant was aspirated and the pellet was then resuspended in sterile 1X Hank’s balanced salt solution (HBSS).

### Exosome quantification

The Zetasizer has the ability to measure particle sizes, but does not give information about quantity of particles. The Nanosight (Malvern) utilizes Nanoparticle Tracking Analysis (NTA), which is a method of visualizing and analyzing particles in a liquid suspension in size range of 10-2000nm. It utilizes Brownian motion by visualizing the particles by virtue of the light that is scattered by them when illuminated by a laser and has a single particle detection system that allows for quantification of particles. Exosomes are quantified using the Nanosight NS300 (Malvern). After being isolated by the above method, they were then diluted 1:10 in sterile HBSS. The samples were pumped at a continuous flow speed value of 20 with a syringe pump. The Nanosight was set to a camera level of 10 and 5 videos at 30 seconds each were recorded. Nanoparticle Tracking analysis (NTA) software (Malvern) was then used to analyze the videos.

### Cell proliferation/viability (MTT) assay

C81-61 cells were plated at a concentration of 5X10^3^ cells per well in a 96-well plate. Cells were treated with R10 or conditioned R10 (RPMI, 10% FBS, 1% Penicillin/streptomycin) media from either C81-61 cells or C81-61-GRM1-6 cells. 10 μl of Thiazolyl Blue Tetrazolum Bromide (Sigma Cat# M5655) in 1XPBS (MTT solution 1) was added on days 1, 3 or 5 to each well, incubated for 4-6 hours at 37°C. MTT solution 2 [(10% Sodium dodecyl sulfate (SDS) in 0.01M HCl)] at an equal volume was added and incubated overnight at 37°C. A 96-well plate reader (Infinite 200, Tecan) was used to measure the absorbance at 550 nm with a reference wavelength of 750 nm.

### Wound healing assay

Conditioned media were collected from either C81-61 (2×10^5^ cells) or C81-61-GRM1-6 (10^5^ cells) that were plated in 60mm plates with R10 for 48 hours at 37°C. For wound healing assays, C81-61 cells (3×10^5^) were plated in a 12-well plate. After 24 hours, the media were replaced with conditioned media, and incubated overnight. Each well was then scratched with a pipette tip, and washed drop-wise with sterile 1XPBS three times, and R10 medium was added. Photographs of the cells (10X, Keyence BZ-X710 microscope) were taken at 0, 4, 6, 12 and 24 hours after media replacement. Area of the wound was calculated using ImageJ and normalized to hour 0.

To test the effects of exosomes on cell migration, exosomes were isolated from the conditioned media as above, and resuspended in R10 medium. C81-61 cells were incubated overnight with the isolated exosomes resuspended in R10 following the same procedure as described above. In order to determine significance, student’s T-test was performed at each time-point, and a p value <0.05 was considered significant.

### Matrigel invasion assay

The matrigel invasion assay previously performed by Higginbotham et al. [38] was followed using our cell model system. C81-61 (GRM1^-^) and C81-61-GRM1-6 (GRM1^+^) cells were plated at 2x10^5^ and 1x10^5^, respectively, in 60mm plates with R10 medium. Once the cells were attached (4-5 hours), media were then replaced with OptiMEM and continued incubation for 48 hours. Media were collected, and exosomes isolated as described above. C81-61 cells were incubated with either GRM1^+^ or GRM1^-^ exosomes for 2 hours, in snap-cap tubes under constant rotation at 37°C. The cells were pelleted and resuspended in serum-free RPMI. A total of 6.5×10^4^ cells were plated on each Corning Matrigel Plate (Corning Cat#354481), which was rehydrated prior to cell plating per instruction by the manufacturer. Exosomes were added to the lower chamber of each well in R10 media, which was replaced every 24 hours. The cells were incubated in the chambers for 72 hours at 37°C, 5% CO_2_. Cells that remained on the top of the chamber were removed by cotton swab, and the membranes were fixed with 100% methanol for 2 minutes, stained with 1% toluidine blue in 10% borax and rinsed with deionized water for 2 minutes. The membranes were dried, detached and mounted on a microscope slide with mounting oil, and an image of the slide was captured under a Keyence BZ-X710 microscope. The stained cells were then counted in 10 random fields of the membrane. Student’s t-test was performed to determine statistical significance.

### Microscopy

The Keyence BZ-X710 microscope was utilized to confirm the presence of either the ptdTomato or GFP fluorescent-tagged proteins when generating stable clones of C81-61 CD63-GFP, C8161 ptdTomato-CD81 and C81-61 ptdTomato- CD81.

### Anchorage-independent assay

Tissue culture 60mm plates were layered with a mixture of 4 ml final concentration of 0.5% agarose in R10. After allowing the agarose on the plates to become solidified for 1-2 hours at 4°C. 1205Lu (as a positive control) or C81-61 CD63-GFP cells were plated at 1x105/plate in 4 ml of final concentration of 0.33% agarose in R10 with exosomes isolated from either C8161 ptdTomato-CD81 or C81-61 ptdTomato-CD81 at 1μl/ml. Fresh R10 medium with 0.33% agarose and exosomes were added once a week for 21 days. The number of colonies in the semi-solid agarose media was counted. The Keyence BZ-X710 microscope was utilized to obtain photographs of the colonies formed in the agarose.
